# Conditioned medium from amniotic cells protects striatal degeneration and ameliorates motor deficits in the R6/2 mouse model of Huntington's disease

**DOI:** 10.1111/jcmm.14113

**Published:** 2018-12-25

**Authors:** Carmela Giampà, Alessandra Alvino, Marta Magatti, Antonietta R. Silini, Antonella Cardinale, Emanuela Paldino, Francesca R. Fusco, Ornella Parolini

**Affiliations:** ^1^ Istituto di Anatomia Umana e Biologia Cellulare, Università Cattolica del Sacro Cuore Rome Italy; ^2^ Fondazione Policlinico Universitario A. Gemelli IRCCS Rome Italy; ^3^ Centro di Ricerca E. Menni, Fondazione Poliambulanza Brescia Italy; ^4^ Laboratory of Neuroanatomy Santa Lucia Foundation IRCCS Rome Italy

**Keywords:** human amniotic mesenchymal stem/stromal cells, Huntington's disease, microglia, neuroinflammation, secretome

## Abstract

Inflammation significantly impacts the progression of Huntington's disease (HD) and the mutant HTT protein determines a pro‐inflammatory activation of microglia. Mesenchymal stem/stromal cells (MSC) from the amniotic membrane (hAMSC), and their conditioned medium (CM‐hAMSC), have been shown to possess protective effects in vitro and in vivo in animal models of immune‐based disorders and of traumatic brain injury, which have been shown to be mediated by their immunomodulatory properties.

In this study, in the R6/2 mouse model for HD we demonstrate that mice treated with CM‐hAMSC display less severe signs of neurological dysfunction than saline‐treated ones. CM‐hAMSC treatment significantly delayed the development of the hind paw clasping response during tail suspension, reduced deficits in rotarod performance, and decreased locomotor activity in an open field test. The effects of CM‐hAMSC on neurological function were reflected in a significant amelioration in brain pathology, including reduction in striatal atrophy and the formation of striatal neuronal intranuclear inclusions. In addition, while no significant increase was found in the expression of BDNF levels after CM‐hAMSC treatment, a significant decrease of microglia activation and inducible nitric oxide synthase levels were observed. These results support the concept that CM‐hAMSC could act by modulating inflammatory cells, and more specifically microglia.

## 1 INTRODUCTION

Huntington's disease (HD) is a fatal, incurable autosomal dominant neurodegenerative disorder caused by instable expansion of polyglutamine (polyQ) tract within the resulting mutated HTT protein (mHTT).^1^ Huntington's disease pathology is characterized by a massive loss of neurons in the striatal part of the basal ganglia^2^ that consequently causes motor and cognitive dysfunction. Although the exact mechanisms by which the polyQ expansion in HTT promotes clinical disease remain incompletely understood, it has been shown that mHTT interferes with several intracellular activities including protein degradation, and mitochondrial respiration and transcription, leading to neuronal malfunction and cell death.

Recent data demonstrate the existence of inflammatory processes in HD pathophysiology. As a matter of fact, there is substantial evidence that the expression of mutant HTT protein results in a pro‐inflammatory activation of microglia which influences disease onset and progression.^3^ More specifically, inflammatory cytokines have been detected in the striatum of HD patients compared with that of healthy individuals^4^ and also in the plasma and cerebrospinal fluid of HD patients.^3^ Furthermore, pro‐inflammatory (M1) microglia relevant biomarkers have been detected in the brains of HD patients, which indicates that M1 microglia may play a crucial role in the pathogenesis of HD.^5^ These findings indicate that central and peripheral inflammation may represent a critical juncture in the progression and amplification of HD pathology that, if understood, could support the development of anti‐inflammatory‐based treatment options.

Indeed, current anti‐inflammatory therapies have been tested in animal models and in patients with HD,^6^ such as XPro595, an inhibitor of TNF‐α^7^ and Laquinimod (NCT02215616), an immunomodulator of activated monocytes.^8^ Although not belonging to the classical definition of anti‐inflammatory drugs, minocycline and cannabinoids have also been tested in HD and have been shown to possess anti‐inflammatory properties in preclinical studies and clinical trials.^9^, ^10^, ^11^ However, the protective effects of minocycline are controversial, despite the fact that minocycline has been reported to be well tolerated and safe,^10^ a worsening of the disease along with detrimental effects, have been reported in animal models^12^, ^13^ and HD patients.^14^

In addition to anti‐inflammatory drugs, the use of cell therapies, and more specifically of mesenchymal stem/stromal cells (MSCs) has become increasingly attractive for neurodegenerative disorders. The ability to work synergistically with the endogenous microenvironment to stimulate resident cell proliferation or neuroprotection via trophic factor secretion, enhances the regenerative potential in injured tissue.^15^ Mesenchymal stem/stromal cells from bone marrow have been used in HD mouse models resulting in a decrease of the striatum atrophy and an increase differentiation of endogenous neural stem cells.^16^ Furthermore, MSCs engineered to overexpress brain‐derived neurotrophic factor (BDNF) were shown to improve motor activity in R6/2 mice.^17^ Brain‐derived neurotrophic factor plays a major role in the survival of mature neurons in the striatum,^18^ and is decreased in brain tissue from HD patients.^19^ Indeed, an ongoing prospective observational study is investigating genetically‐engineered MSC to secrete BDNF in HD patients (PRE‐CELL: Clinicaltrials.gov identifier NCT01937923),^20^ and subsequently a Phase I trial will be performed to assess the safety of genetically‐modified MSCs to treat HD in patients.

In the context of MSC, those derived from the amniotic membrane of human term placenta (hAMSC) have attracted much attention for several reasons. First, the use of placenta as a cell source bypasses the problem of the availability of tissue source since placenta is normally discarded as medical waste after birth, and it is easy to obtain without ethical controversy. Second, hAMSC possess strong immunomodulatory properties and transplantation of hAMSC has repeatedly shown to favour tissue repair and regeneration in rodent models of inflammatory‐based diseases.^21^ Similar benefits were achieved when cell‐free treatments were used, such as conditioned media containing cell‐secreted factors during in vitro culture.

Bioactive factors produced by amniotic cells (ie secretome) are able to act on different inflammatory mediators in vitro. More specifically, the hAMSC secretome has been shown to induce anti‐proliferative effects on T cells, skew T cell polarization by enhancing T regulatory cells and reducing Th1 and Th17 populations, and inhibit the differentiation of monocyte‐derived dendritic cells.^22^ Of particular interest is a recent finding that hAMSC and conditioned medium from hAMSC (CM‐hAMSC) induced comparable protection in in vitro and in vivo models of traumatic brain injury.^23^ More specifically, they enhanced neuronal rescue, decreased pro‐inflammatory M1 and promoted M2 microglia polarization, and induced neurotrophins involved in neuronal and vascular remodelling (BDNF and vascular endothelial growth factor, VEGF). In addition to BDNF, other neurotrophins such as nerve growth factor (NGF) and neurotrophin‐3 (NT‐3) have been reported to be expressed by amnion.^24^ Furthermore, metabolomic profiling has shown that CM‐hAMSC contains lysine, taurine, alpha‐aminoadipic‐acid and spermidine,^23^ all of which have been reported to possess neuroprotective effects.^25^, ^26^, ^27^, ^28^, ^29^ Conditioned medium from hAMSC has also been reported to contain anti‐inflammatory molecules^22^ and various factors implicated in the growth, differentiation, vascularization and survival of neurons and synapses, including interleukin 10 (IL‐10), transforming growth factor beta (TGF‐β),^30^ hepatocyte growth factor (HGF),^31^ prostaglandin E2 (PGE2),^32^ angiogenin^33^ and leptin.^34^

At present, there are few options to treat the symptoms of HD and no therapy to slow the neurodegenerative process. Here, we hypothesize that the immunomodulatory actions and the trophic factors in CM‐hAMSC could have beneficial effects in a mouse model of HD, thus supporting the potential development of a cell‐free therapy. The purpose of this study was to assess a novel therapeutic intervention with CM‐hAMSC in symptomatic HD mice, and investigate if CM‐hAMSC ameliorates the behavioural and neuropathological sequel of mutant huntingtin (mHtt).

## 2 MATERIALS AND METHODS

### 2.1 Isolation of hAMSC and preparation of CM

Human term placentae were collected at the Department of Obstetrics and Gynecology of Fondazione Poliambulanza hospital in Brescia from healthy women after vaginal delivery or caesarean section at term. Samples were collected after obtaining informed written consent according to the guidelines set by the *Comitato Etico Provinciale* of Brescia, Italy. hAMSC were isolated from the amniotic membrane using well‐established techniques.^35^ Conditioned medium from hAMSC was generated by culturing hAMSC for 5 days in 24‐well plates (Corning Inc, Corning, NY) (0.5 × 10^6^ cells/well in a final volume of 0.5 mL), in serum‐free Neurobasal (NB) medium supplemented with B27 (both from Life Technologies, Monza, Italy) (NB/B27, B27 1:50; L‐glutamine, 1:100; penicillin, 100 U/mL; streptomycin, 100 μg/mL). The medium used as a control (CTRL) was NB/B27 cultured for 5 days. The supernatants were collected, centrifuged, filtered and stored at −80°C until use.^23^

### 2.2 Animal model and CM‐hAMSC administration

All studies were conducted in accordance with European Communities Council Directive of 24 November 1986 (86/609/EEC) as adopted by the Santa Lucia Foundation Animal Care and committee. R6/2 (B6CBATg(HDexon1)62Gbp/1J) mice, which express exon 1 of the human mutant HD gene containing 160 ± 5 CAG repeat expansions, under the control of the human *HTT* (IT15) promoter, were obtained by crossing ovarian transplanted hemizygous females with B6BAF1/J males (Jackson Laboratories #002810). To limit possible variations in the phenotype of R6/2 mice due to CAG repeat size,^36^ all experiments were conducted on the first offspring in which the number of CAG repeat length varies very little and can be considered to be approximately 160 CAG (http://chdifoundation.org/wp-content/uploads/HD_Field_Guide_040414.pdf). The offspring were genotyped by PCR assay following the JAX standard protocol, using the following primers: 5ʹ‐CCG CTC AGG TTC TGC TTT TA‐3ʹ and 5ʹ‐GGC TGA GGA AGC TGA GGA G‐3ʹ. Using these primers, we confirmed that all mice used in this study had approximately 160 ± 10 CAG repeat with corresponding base pairs of 600, as determined by PCR.

Mice were weaned and treatments began at 5 weeks of age when mice were fully symptomatic. The study groups were: wild‐type (WT) or R6/2 mice treated with saline, CTRL or CM‐hAMSC. Animals were given daily intraperitoneally (ip) injections with 150µL of saline, CM‐hAMSC or CTRL, 6 days a week for 9 weeks. The number of animals used is shown in Table 1. Mice were handled by the same investigator at the same time every day. Mice were identified by a randomly assigned code and housed five per cage under standard conditions with ad libitum access to food and water. Data were collected by observers who were blinded to treatment.

**Table 1:** Number of animals used for the study

WT + saline n = 37	WT + CM‐hAMSC n = 6	WT + CTRL n = 6
R6/2 + saline n = 16	R6/2 + CM‐hAMSC n = 21	R6/2 + CTRL n = 18

### 2.3 Assessment of neurological and behavioural function

#### 2.3.1 Body weight assessment

Animals were weighed daily starting at the beginning of treatment and the average weekly weight was calculated. Since male and female R6/2 mice show distinct weight gain and loss rates,^37^ changes in the body weights of male and female mice within each group were analysed separately. The criterion for euthanasia was the point in time when mice could no longer stand after being placed on their side for 30 seconds, according to Stack and co‐workers.^38^

#### 2.3.2 Analysis of motor coordination (rotarod)

Motor coordination and balance were estimated using a five‐station mouse rotarod (Rotarod/RS LSI Letica, Biological Instruments, Varese, Italy). Four‐week old mice were trained at increasing speeds up to a constant speed of 14 rpm for three consecutive trials. Subsequently, they were administered one rotarod trial twice a week from 5 to 13 weeks of age. Mice were given three trials on the rod, and their latencies to fall were measured and averaged. A maximum latency of 60 seconds was defined.

#### 2.3.3 Analysis of motor activity (open field)

Motor activity was measured in an open field consisting of a circular arena (60 cm diameter) with the floor divided into central and peripheral sectors by black lines. Mice were placed in the arena for 10 minutes during which the distance travelled was recorded by means of dedicated software (Noldus, Wageningen, the Netherlands).

#### 2.3.4 Clasping

When suspended by the tail, R6/2 mice exhibit a hind‐limb clasping phenotype that indicates neurological impairment.^39^ Mice were suspended by their tail for 60 seconds. The total amount of time spent clasping was recorded twice a week and the average clasping duration per week was calculated.

### 2.4 Morphological studies

Under deep anaesthesia mice were transcardially perfused with saline solution followed by 60 mL of 4% paraformaldehyde in saline solution. Brains were removed, collected and post fixed in 4% paraformaldehyde overnight. The following day, brains were cryoprotected in 10% sucrose and 20% glycerol in 0.1 mol/L phosphate buffer with sodium azide 0.02% for 48 hours at 4°C. Frozen brains were subsequently sectioned at a 40 μm‐thickness using a sliding microtome.

#### 2.4.1 Analysis of striatal volume

Coronal step serial sections from rostral neostriatum through the level of anterior commissure (from bregma 1.54 to bregma −0.22, with an interval of 120 µm between sections) were employed and stained with an antibody against Calbindin D‐28 K, a marker of the Medium Spiny Neurons (dilution 1:500, CALB, Immunological Sciences). Immunoreactivity was visualized using diaminobenzidine‐immunoperoxidase. The striatal area was traced in each section and the gross striatal volume was measured using Stereo Investigator software (Zeiss, Cochester, VT, USA) and the Cavalieri Estimator probe.

#### 2.4.2 Evaluation of neuronal intranuclear inclusion

Sections were processed for single label EM‐48 mHtt protein (mouse anti‐EM48 1:500, Immunological Science, Rome, Italy) by means of immunofluorescence and were counterstained with NeuroTrace (a neuronal marker, 1:200, Chemicon, USA). A sample of approximately 250 neurons per hemisphere for each of three sections, in each of six mice per treatment group, was analysed to determine the number and the area of intranuclear mHTT aggregates in striatal neurons. Images were acquired with a 63X objective on a confocal laser scanning microscopy (CLSM) (Zeiss LSM 700) laser‐scanning confocal microscope under nonsaturating exposure conditions. The same acquisition settings were used for all samples. Images were analysed using the Java image processing and analysis program Image j, developed by Wayne Rasband, available at http://imagej.nih.gov/ij/docs/index.html.

### 2.5 Analysis of microglial activation

Activated microglia were detected in the striatum by immunolabelling with rat anti‐mouse CD68 antibody (1:300; Immunological Science). For each mouse, three coronal brain, rostrocaudally spaced, 40 µm‐sections of the striatum (+1,18, +0,86, +0,38 mm from bregma, KBJ Franklin and G Paxinos, The Mouse Brain in Stereotaxic Coordinates, Academic Press), were used to quantify the percentage of CD68 immunostained area. For each section, the striatum was subdivided in six representative fields using 40× magnification (the total area of each field analysed was 0.050625 mm^2^). A schematic representation of the regions of interest and the selected fields is depicted in Figure 5A. Images were acquired using a CLSM (Zeiss LSM 700) laser‐scanning confocal microscope under nonsaturating exposure conditions and using the same acquisition settings for all samples. The conditions in terms of gain and laser power were selected at levels that allowed optimal visualization of the fluorophore used as secondary antibody and standardized using sections from wild‐type mice. Each image was saved at a resolution of 1024 × 1024 pixels. These settings were applied as standard for subsequent images. Using a 40× objective, Z‐stacks images of striatum from coronal sections were collected using computer controlled microstepper stage of the confocal microscope. Images stacks were combined into a single two‐dimensional (2D) projection image, exported in TIF file format using NIH ImageJ software and, using the adjusted threshold function, the area of CD68 positive tissue was quantified. The CD68 immunostained area was calculated as CD68 immunostained area/the total area analysed and indicated as staining percentage area.

### 2.6 Western blotting

Protein lysates were obtained from the striatum by homogenization (50 mmol/L Tris HCl, pH 7.5; 150 mmol/L NaCl; 1% Triton X‐100; 5% glycerol; 1% sodium deoxycholate; 0.1% SDS; 5 mmol/L: ethylenediaminetetraacetic acid; 1 mM EGTA; 1X protease inhibitor cocktail), followed by sonication and centrifugation. Protein quantification was performed with the Bradford method (Bio‐Rad, USA) and 50 µg of protein were separated on a SDS‐PAGE gel and then transferred onto PVDF membranes (Amersham Biosciences, Italy). Membranes were blocked with 5% non‐fat dried milk and incubated overnight at +4°C in 3% non‐fat dried milk with the primary antibody (rabbit anti‐BDNF 1:1000, Immunological Sciences; rabbit anti‐iNOS 1:1000, Immunological Sciences; mouse anti β‐actin 1:20000, Sigma, USA), and the appropriate secondary antibody (HRP 1:5000, Invitrogen, USA) for 2 hours. Bands were detected with the Clarity ECL (Bio‐Rad) and the Chemidoc Touch Imaging System (Bio‐Rad) and analysed with Imagej software.

### 2.7 Statistical analysis

The data collected were analysed to compare the effect of CM‐hAMSC on behavioural and neuropathological outcomes of the different treatment groups. Statistical analysis was performed using either a one‐way or a two‐way repeated measures ANOVA followed by HSD Tukey test. *P* values less than 0.05 were considered to be statistically significant.

## 3 RESULTS

### 3.1 CM‐hAMSC treatment does not affect weight loss in R6/2 mice

**Figure 1:**
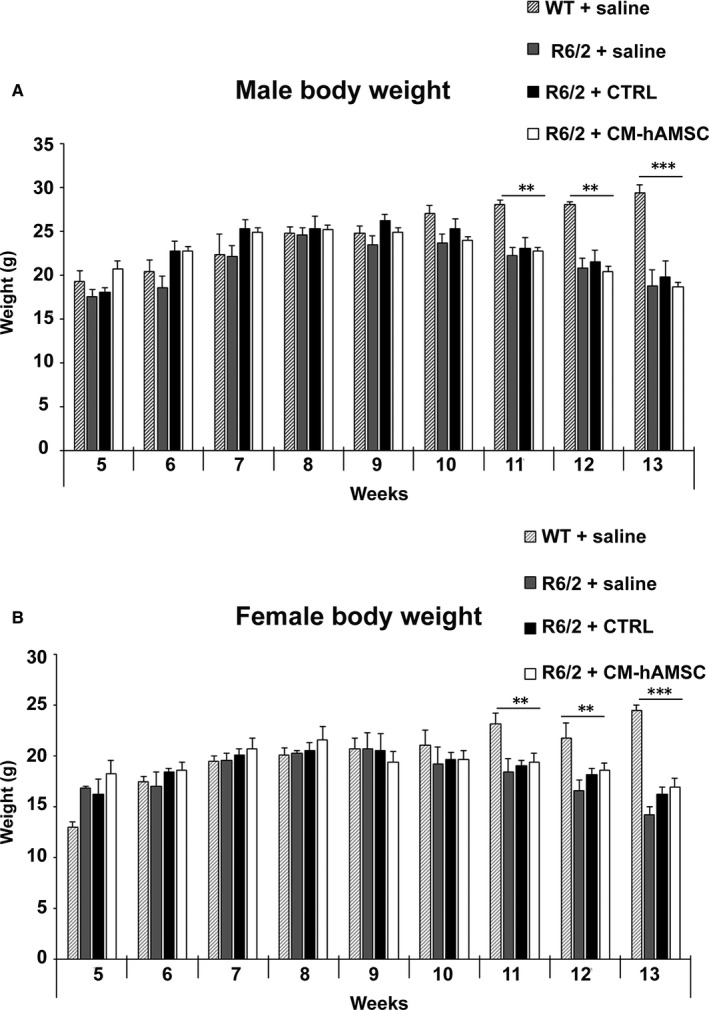
Effects of conditioned medium from hAMSC (CM‐hAMSC) on the body weights of R6/2 mice. Body weights of (A) male and (B) female wild‐type (WT) mice treated with saline, and R6/2 mice treated with saline, control medium (CTRL) or CM‐hAMSC. Data are presented as mean + SD. There was no significant effect of CM‐hAMSC on the body weight of male and female R6/2 mice; **P < 0.01 and ***P < 0.001 vs WT + saline

Progressive loss of body weight is a consistent and robust feature of R6/2 mice.^36^ When compared to wild‐type mice treated with saline, there was a significant decline of body weight in both female and male R6/2 mice at 11 (*P* < 0.01), 12 (*P* < 0.01) and 13 (*P* < 0.001) weeks of age which represents the time at which R6/2 mice manifest clinical symptoms (Figures 1A,B). In both genders, we found no significant effect of CTRL or CM‐hAMSC on body weight loss of R6/2 mice (Figure 1A,B).

### 3.2 The onset of the clasping reflex is ameliorated in R6/2 mice treated with CM‐hAMSC

**Figure 2:**
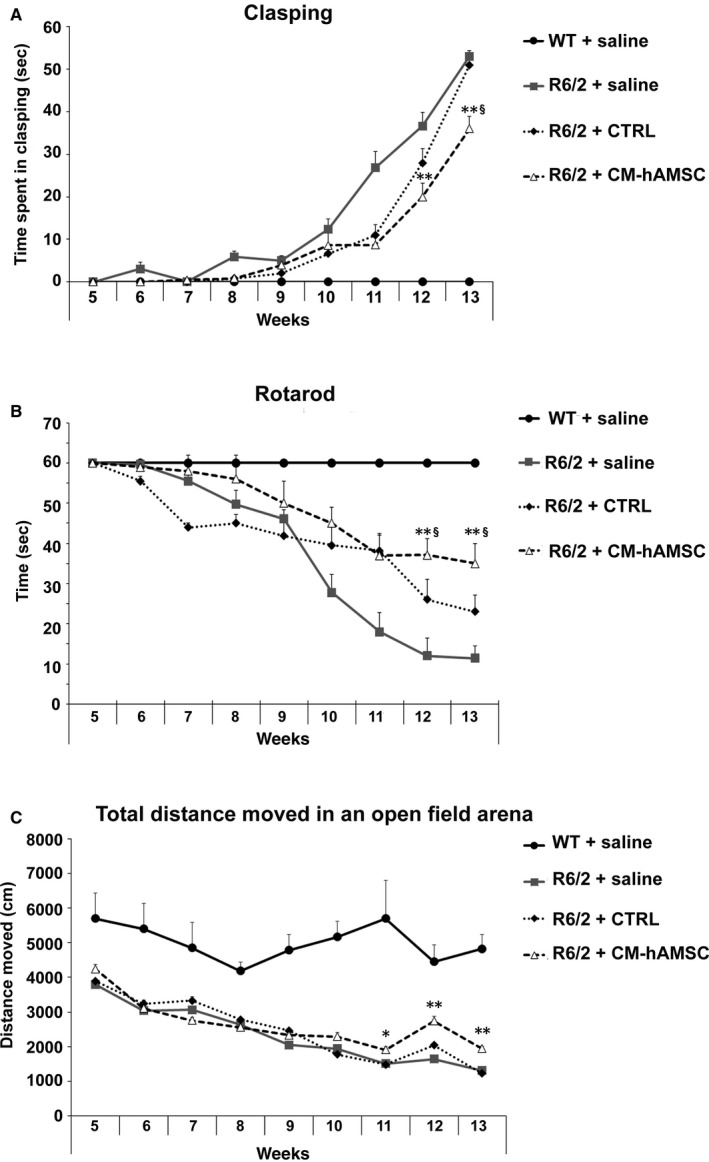
Conditioned medium from hAMSC (CM‐hAMSC) treatment restores motor symptoms in R6/2 mice. A, The development of the hind‐limb clasping phenotype in wild‐type (WT) mice treated with saline, and R6/2 mice treated with saline, control medium (CTRL) or CM‐hAMSC. One way ANOVA with repeated measurements indicated a statistically significant effect of treatment with CM‐hAMSC (*P* < 0.00002) at 12 and 13 weeks of age versus R6/2 treated with saline. B, Latency to fall from the accelerating rotarod in the R6/2 mice. A one way ANOVA indicated that R6/2 mice treated with saline exhibited a progressive decrease in the latency to fall and this decrease was blunted by CM‐hAMSC treatment. C, Total distance travelled during a 10 min test session in an open field test. The CM‐hAMSC treated R6/2 mice had a lesser decrease in distance travelled as compared to the saline treated R6/2 mice in the late stage (12‐13 weeks of age) of disease (*P* < 0.00002). *vs R6/2 + saline; § vs R6/2 + CTRL

The clasping reflex, an abnormal posturing of the hind limb during the tail suspension, is a typical phenotype alteration characteristic of R6/2 mice^36^ that reflects the progression of brain damage. Clasping is absent in wild‐type mice. In saline‐treated R6/2 mice, the clasping response was evident by 9 weeks of age and then developed progressively and reached the maximal levels by 13 weeks of age, when the mice are full symptomatic (Figure 2A). R6/2 + CTRL steadily increased their clasping behaviour starting from 11 weeks of age until 13 weeks of age, at which time there was no difference compared to R6/2 mice treated with saline. In contrast, the clasping response developed more gradually in R6/2 mice treated with CM‐hAMSC. Indeed, the time spent exhibiting the clasping response was significantly less in the CM‐hAMSC‐treated mice compared to saline‐treated R6/2 mice at 12 and 13 weeks of age (Figure 2A, *P* < 0.00002). Moreover, at 12 and 13 weeks of age when fully symptomatic, R6/2 mice treated with CM‐hAMSC showed less clasping compared to mice treated with CTRL (Figure 2A, *P* < 0.00002). These data indicated that CM‐hAMSC was highly effective in attenuating clasping behaviour detected in a response that reflects dystonia.

### 3.3 R6/2 mice treated with CM‐hAMSC show improvements in motor coordination

To further characterize the effect of CM‐hAMSC treatment on behavioural phenotype, we tested motor coordination and balance by rotarod analysis. Mice were trained to maintain balance on an accelerating rotarod beginning at 5 weeks of age and then tested weekly until 13 weeks of age. Motor coordination was severely impaired in saline‐treated R6/2 mice in a time‐dependent manner compared to WT mice (significant effect of time: F(5.79) = 51.37; *P* < 0.0001). Conditioned medium from hAMSC affected performance in a group‐dependent fashion (significant group × time interaction: F(40.632) = 18.89; *P* < 0.0002). There was a significantly higher performance in CM‐hAMSC‐treated R6/2 mice than the saline‐ or CTRL‐treated R6/2 mice at 12 and 13 weeks of age, suggesting that, the CM‐hAMSC‐treated R6/2 mice advanced with significantly less severity in the late stage of HD (Figure 2B).

### 3.4 CM‐hAMSC treatment ameliorates motor activity

Finally, to confirm the improvement in motor performance and to study the exploration levels, we performed the open field test. Motor activity data collected in the open field test indicated that R6/2 mice showed a progressive decrease in spontaneous locomotor and exploratory activity in the arena when compared with wild‐type mice (Figure 2C; group effect for total distance moved F(3.87) = 35.28; *P* < 0.0001). This significant increase to hypoactivity was equally observed in R6/2 mice treated with CTRL medium as there were no statistically significant differences between saline‐ or CTRL‐treated R6/2 mice (Figure 2C). The CM‐hAMSC‐treated R6/2 mice travelled a significantly greater distance than the vehicle‐treated R6/2 animals in the late stage of disease (from 11 weeks up to 13 weeks of age (*P* < 0.00002, Figure 2C).

For all three motor activity assessments (clasping, rotarod, open field) no differences were observed between WT mice treated with saline, CTRL medium and CM‐hAMSC (data not shown).

### 3.5 Improvement of neuropathological assessments of R6/2 mice after treatment with CM‐hAMSC

Since CM‐hAMSC‐treated R6/2 mice showed greater preservation of their striatal‐dependent motor coordination than saline‐ or CTRL‐treated R6/2 mice, we next evaluated whether striatal neuropathology correlated with these behavioural results.

#### 3.5.1 Striatal volume

**Figure 3:**
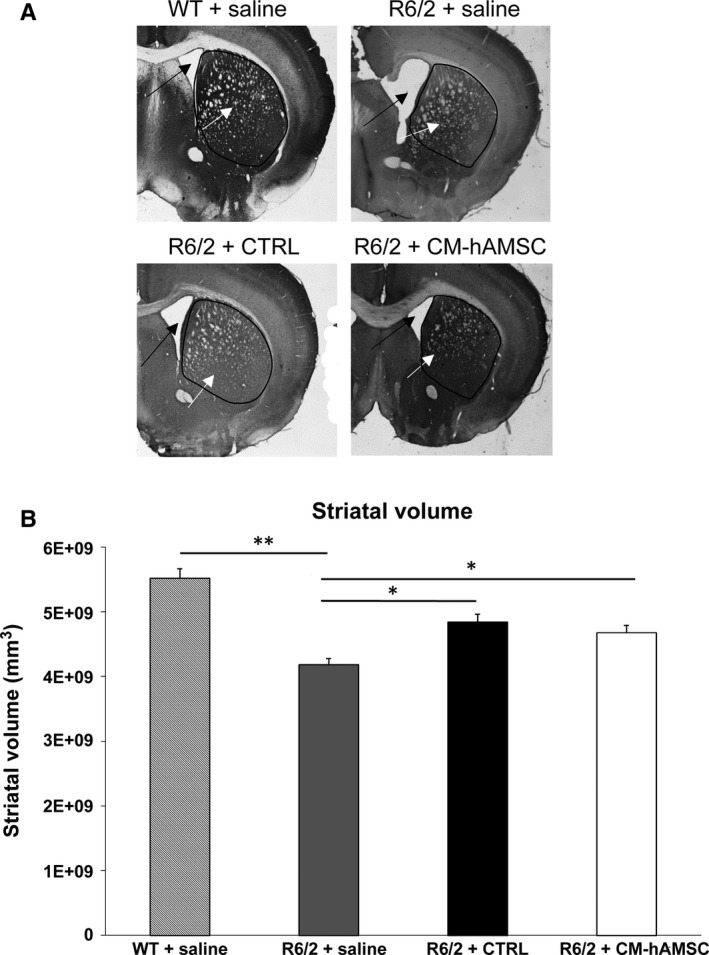
Effects of Conditioned medium from hAMSC (CM‐hAMSC) treatment on striatal atrophy in R6/2 mice at 13 weeks of age. A, Transmitted light microscope images showing representative calbindin‐stained coronal sections of a wild‐type (WT) mice treated with saline, and saline‐, CTRL‐ or CM‐hAMSC‐treated R6/2 mice. Marked gross striatal atrophy (black arrows) and enlarged lateral ventricles (white arrows) are present in vehicle‐treated R6/2 mice compared to wild‐type mice. This atrophy is mostly absent from the sections of the R6/2 mouse treated with CTRL or CM‐hAMSC. B, Quantification of differences in striatal volume in the same groups as (A). Post hoc analysis indicated that R6/2 mice treated with saline had a significantly reduced striatal volume compared to the wild‐type group (*P < 0.05; **P < 0.01)

Striatal atrophy in R6/2 mice was assessed by measuring striatal areas in the calbindin‐stained brain sections in 13‐week old mice (Figure 3). Figure 3A shows representative microphotographs of coronal sections immunostained for calbindin. The area of the striatum was significantly reduced in brain sections from saline‐treated R6/2 mice compared with those from saline‐treated WT mice (*P* < 0.01; Figure 3B). Repeated treatment with CM‐hAMSC significantly inhibited the decline of striatal area in R6/2 mice (*P* < 0.05; Figure 3B). Unexpectedly, the treatment with the CTRL was also able to reduce neurodegeneration in the striatum of R6/2 mice and there was no difference when compared to CM‐hAMSC‐treated R6/2 mice.

#### 3.5.2 mHtt aggregation

**Figure 4:**
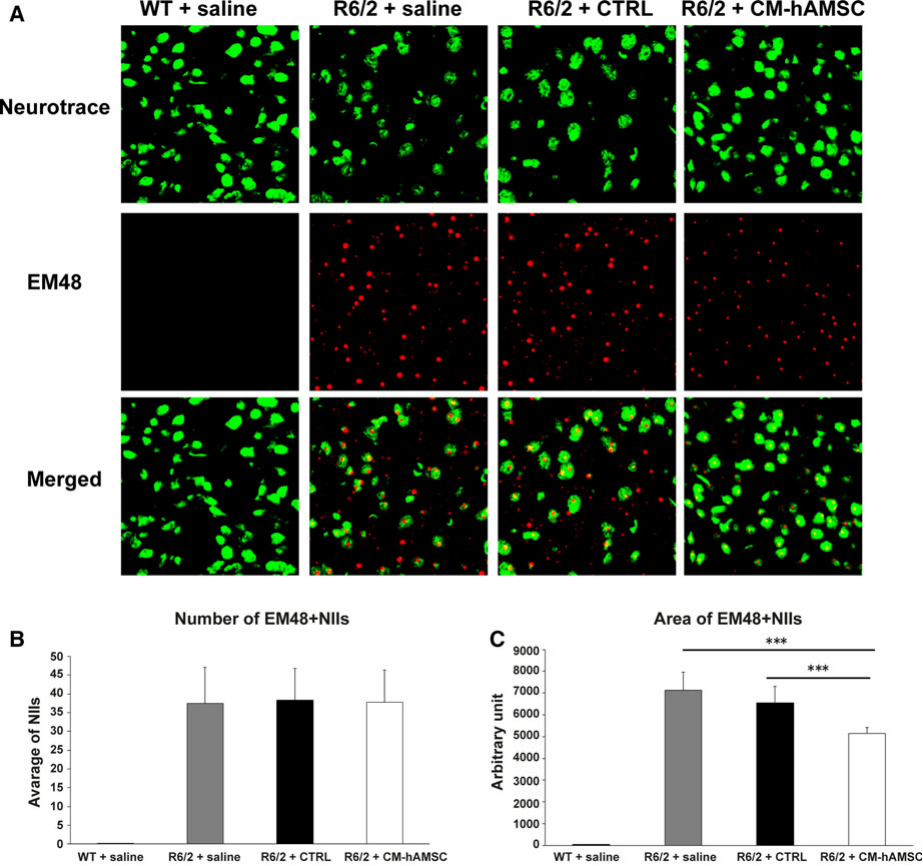
Double‐label immunofluorescence for Neurotrace and neuronal intranuclear inclusions (NIIs). A, The double label immunostaining was performed in wild‐type mice, and in saline‐, CTRL‐ and conditioned medium from hAMSC (CM‐hAMSC)‐treated R6/2 mice. Neurotrace was used to label all neurons in the striatum and EM48 to stain the intranuclear NIIs (visualized in red fluorescence). Neuronal intranuclear inclusions were absent in wild‐type mice. (B,C) Graphs show the effect of CM‐hAMSC on NIIs number and size. The one‐way repeated measures ANOVA performed on data obtained by each group revealed a statistically significant effect of treatment on NIIs size (C, *** *P* < 0.0001)

The expression of exon 1 of mHtt in R6/2 mice results in the formation of neuronal intranuclear inclusions (NIIs) detected with the antibody EM‐48.^40^ Thus, we sought to determine if the improved motor performance observed in CM‐hAMSC‐treated R6/2 mice is associated with attenuation of mHtt aggregate formation. Double immunofluorescence with anti‐Htt antibody EM48 and Neurotrace was performed (Figure 4A). R6/2 mice treated with CM‐hAMSC did not exhibit a decrease in the number of intranuclear aggregates of mHTT (NIIs) as there was no difference between the R6/2 mice treated with saline and both CM‐hAMSC‐ or CTRL‐treated R6/2 (*P* > 0.05; Figure 4B). However, when we measured the area of NIIs, we found a marked reduction in mHtt size in R6/2 treated with CM‐hAMSC when compared with saline‐ (*P* < 0.0001) or CTRL‐treated (*P* < 0.0001) R6/2 mice, thus indicating that the treatment with CM‐hAMSC was able to reduce the aggregation of mutant Huntingtin (Figure 4C).

### 3.6 CM‐hAMSC protects R6/2 mice against neuroinflammation

Due to the well‐documented immunomodulatory properties of CM‐hAMSC, we asked whether the beneficial effects of conditioned medium could be associated with an anti‐inflammatory effect exerted in the striatum of R6/2 mice model. Thus, we investigated the expression of CD68 (marker for activated microglia) and iNOS (pro‐inflammatory, M1 marker).^41^

#### 3.6.1 CM‐hAMSC treatment reduces reactive microglia in the striatum of R6/2 mice

**Figure 5:**
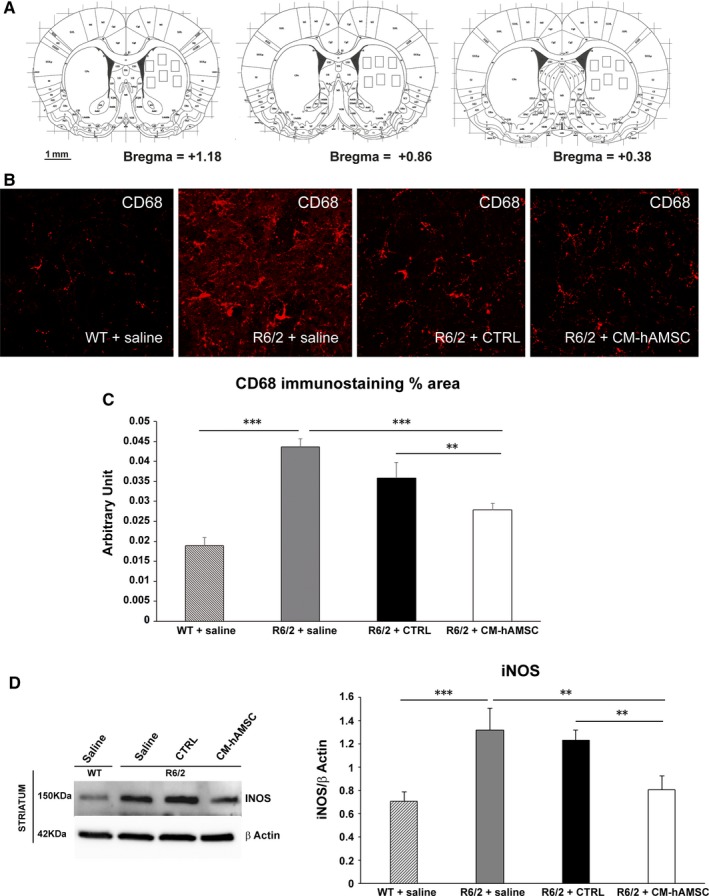
Conditioned medium from hAMSC (CM‐hAMSC) treatment attenuates neuroinflammation in R6/2 mice. A, Squares represent areas of tissue sampling for quantification of CD68 immunostaining. B, Confocal laser scanning microscopy (CLSM) images of single‐label immunofluorescence for CD68 (visualized in red‐Cy3 fluorescence) in the striatum region from wild‐type mice, R6/2 mice treated with saline, CTRL and CM‐hAMSC. C, Histogram showing the microglial reactivity quantification by NIH software. Data are presented as the mean values of CD68 positive areas ±SEM, (**P < 0.001; ***P < 0.001). D, M1 macrophage marker inducible nitric oxide synthase (iNOS) protein levels were evaluated by Western Blot in striatal homogenates from wild‐type mice (WT) and saline‐, control medium (CTRL)‐ and CM‐hAMSC‐treated R6/2 mice. Protein levels were normalized against the expression of beta‐actin. Protein blot signal intensity analysis performed by Imagej software is represented in the graphs (**P < 0.01; ***P < 0.001)

To evaluate microglia reactivity, we performed CD68 immunostaining in the striatum (Figure 5A) and quantified the surface covered by CD68 (Figure 5B,C).^42^ Immunostaining for CD68 in saline‐treated R6/2 mice group revealed an intense microglial reaction, where microglial cells positive for CD68 were numerous and the area of the reaction was higher compared to that observed in wild‐type mice (Figure 5C, *P* < 0.001). Microglial reactions were significantly attenuated in R6/2 mice treated with CM‐hAMSC when compared to R6/2 mice treated with either saline (Figure 5C, *P* < 0.001) or CTRL (*P* < 0.01). These data show that the activation of microglia persists in the late stage of disease when the animals are fully symptomatic, and that treatment with CM‐hAMSC is able to decrease microglia activation.

#### 3.6.2 CM‐hAMSC treatment reduces iNOS levels in the striatum of R6/2 mice

We investigated inflammatory markers that could potentially be involved in the decrease of CD68 after CM‐hAMSC treatment. More specifically, we evaluated the expression of the inducible isoform of nitric oxide synthase (iNOS), a well‐established source of nitric oxide during inflammation of the central nervous system (CNS) and considered a functional marker of M1 phenotype microglia.^41^ Evidence of increased iNOS expression has been observed in HD animal models.^43^ In line with these findings, we found an elevated expression of iNOS in the striatum of saline‐treated R6/2 mice when compared with wild‐type mice (Figure 5D, *P* < 0.001). Moreover, levels of iNOS were significantly reduced by treatment with CM‐hAMSC compared to both saline‐ and CTRL‐treated R6/2 mice (Figure 5D, *P* < 0.01).

### 3.7 CM‐hAMSC treatment does not alter BDNF expression in the striatum of R6/2 mice

**Figure 6:**
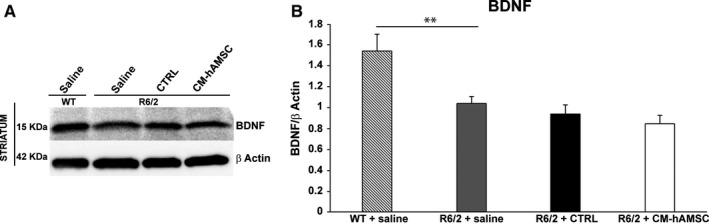
Conditioned medium from hAMSC (CM‐hAMSC) treatment does not alter BDNF protein in the striatum of R6/2 mice. BDNF levels were evaluated by immunoblotting in striatal homogenates from wild‐type mice (WT) and saline‐, control medium (CTRL)‐ and CM‐hAMSC‐treated R6/2 mice. Protein levels were normalized against the expression of beta‐actin. Protein blot signal intensity analysis performed by ImageJ software is represented in the graphs (**P < 0.01)

Brain‐derived neurotrophic factor is one of the principal mediators whose deficiency contributes to the pathology caused by mutant Huntingtin in mice and in HD patients.^18^ Thus, we investigated the effects of CM‐hAMSC on BDNF expression in the striatum of R6/2 mice. Quantification by Western blot showed a significant decrease of BDNF in R6/2 mice treated with saline when compared with wild‐type mice (Figure 6A,B; *P* < 0.01). Treatment of R6/2 mice with CM‐hAMSC was not able to revert the decrease in BDNF, suggesting that the beneficial effects of CM‐hAMSC were not be mediated by an increase of BDNF levels in the striatum.

## 4 DISCUSSION

Herein, for the first time, we have tested a cell‐free treatment based on CM‐hAMSC in the R6/2 mouse model of HD. Our findings demonstrate that peripheral administration of CM‐hAMSC has a therapeutic effect on the behavioural impairment in the R6/2 mouse. Indeed, CM‐hAMSC was effective in reinstating motor performance in R6/2 mutants, as assessed by the clasping, open field and rotarod tests. In line with the significant delay of the development of neurological impairment, CM‐hAMSC treatment also ameliorated neuropathological changes in R6/2 mice, including the decrease of striatal volume and of neuronal intranuclear inclusions, and the reduction of microglia activation.

A reduced intranuclear aggregation of mHtt protein, a typical neuropathological feature of HD, is associated with improved cognition and/or motor performance in HD mice.^44^ Accordingly, we found that the size of the densely stained intranuclear aggregates appeared significantly diminished in CM‐hAMSC‐treated R6/2 mice compared with saline‐ or CTRL‐treated R6/2 mice. In addition, we observed that the reduced intranuclear aggregation of mHtt protein was associated with an improvement of striatal atrophy in CM‐hAMSC‐treated R6/2 mice. Even if the CTRL medium was also able to partially increase striatal volume and transiently ameliorate clasping and rotarod performance, CM‐hAMSC administration was most efficient in both rescuing motor abnormalities and improving neuropathological changes in late stage disease. The transient amelioration observed in CTRL‐treated R6/2 mice could be related to the presence of neurotrophic factors in control media; these factors include progesterone, linoleic and linolenic acid, and L‐carnitine, all of which have been described to exert neuroprotective actions due to their roles in the regulation of cell proliferation, metabolism, stress responses, apoptosis and ageing.^45^, ^46^, ^47^, ^48^

Although it is widely reported that BDNF, an important neuroprotective factor, is reduced in HD patients and in HD animal models^19^, ^49^ the beneficial effect of CM‐hAMSC observed in this study could be BDNF‐independent. Indeed, we did not observe any increase of BDNF levels in the striatum of R6/2 mice treated with CM‐hAMSC. Our data are in line with recent evidence showing that improvement of HD symptoms in the R6/1 mouse model did not correlate with an increase in BDNF.^50^ In addition to a reduction of BDNF, a hallmark of HD that exacerbates neurodegeneration and neurological symptoms is the pro‐inflammatory activation of microglia induced by mHtt aggregates.^51^ In this regard, our findings indicate that CM‐hAMSC‐treatment reduced the aggregation of mHtt and reduced the area of activated microglia in the striatum of R6/2 mice.

Importantly, we observed a reduction of inducible nitric oxide synthase (iNOS) levels in the striata of CM‐hAMSC‐treated mice. The enzyme iNOS is commonly up‐regulated in neurodegenerative diseases including Alzheimer's disease, Parkinson's disease,^52^ and HD.^43^, ^53^, ^54^ Inducible nitric oxide synthase activation has been shown to contribute to oxidative neuronal death in R6/2 transgenic HD mice,^54^ and it is primarily found in microglia with an M1 phenotype.^41^ In line with our observations, other studies have shown that a decrease of iNOS delays disease progression in preclinical models of HD.^53^, ^55^, ^56^ The reduction of iNOS expression and microglia activation could be explained by the presence of anti‐inflammatory molecules,^22^ including IL‐10 and TGF‐β^30^ in CM‐hAMSC. In addition, amnion has been shown to express neurotrophic factors such as BDNF, NGF, and NT‐3,^24^ and CM‐hAMSC contains lysine, taurine, alpha‐aminoadipic‐acid, and spermidine,^23^ HGF, PGE2, angiogenin and leptin,^22^ all of which have been reported to possess neuroprotective effects.^25^, ^26^, ^27^, ^28^, ^29^, ^30^, ^31^, ^32^, ^33^, ^34^

Mesenchymal stem/stromal cells have become increasingly important for the development of cell‐based therapeutics for the treatment of HD.^57^, ^58^ However, studies applying bone marrow or umbilical cord MSC locally in the R6/2 mouse model of HD have shown differing results, such as those that did not observe improvement of motor activity^59^, ^60^ or increase in striatal volume.^60^ On the other hand, others have shown improvements in behaviour, reduced striatal degeneration, and reduced ubiquitin‐positive aggregates in R6/2 mice after treatment with adipose tissue MSC.^61^ For these studies, MSC were administered locally in the brain, while currently there is no evidence of MSC beneficial effects when administered systemically. In our study, CM from amniotic MSC was administered systemically thus providing a significant advantage for clinical translation. Moreover, to date, only one other study has demonstrated beneficial effects with a cell (MSC)‐free treatment after systemic administration in the R6/2 mouse model.^62^ In this study, the authors show that repeated intraperitoneal administration of extract from adipose tissue MSC improved performance in the rotarod test, and ameliorated striatal atrophy and mHtt aggregation in the striatum.^62^ These results are similar to our observations herein, and in addition, we show the beneficial effect of CM‐hAMSC on microglia activation. While the mechanism underlying the beneficial properties of CM‐hAMSC remains unclear and deserves further investigation, the peripheral administration of CM‐hAMSC, and its immunomodulatory properties, suggest that the effect of CM‐hAMSC on the peripheral immune system could indirectly influence the CNS. In support of this hypothesis evidence has shown the existence of a bidirectional communication between the injured brain and the peripheral immune system.^63^, ^64^

In conclusion, in this study we show for the first time that peripheral administration of a cell‐free treatment from amniotic MSC is sufficient to induce a protective effect in the CNS of an animal model for HD.

Even though the immunomodulatory properties of CM‐hAMSC, and the bioactive factors implicated in these effects, remain to be clarified, this study reinforces the evidence of the therapeutic properties of hAMSC derivatives in diseases with aberrant inflammatory processes, and in particular opens a novel scenario for a cell‐free therapeutic strategy in HD.

## CONFLICT OF INTEREST

None.
